# What’s in an App? Scoping Review and Quality Assessment of Clinically Available Hearing-Aid-Connected Apps

**DOI:** 10.3390/audiolres15060157

**Published:** 2025-11-13

**Authors:** Kate Pfingstgraef, Robin O’Hagan, Jana N. Bataineh, Danielle Glista

**Affiliations:** 1 School of Communication Sciences and Disorders, Western University, London, ON N6G 1H1, Canada; kpfingst@uwo.ca (K.P.); jbataine@uwo.ca (J.N.B.); 2 National Centre for Audiology, Western University, London, ON N6G 1H1, Canada; rohagan6@uwo.ca; 3 Health and Rehabilitation Sciences, Faculty of Health Sciences, Western University, London, ON N6G 1H1, Canada

**Keywords:** hearing aids, mobile health, mHealth, hearing-aid-connected apps, mobile application rating scale, audiology, app quality

## Abstract

**Background/Objectives:** Mobile health (mHealth) tools, such as smartphone apps, support person-centred care for persons with hearing loss engaging in the hearing aid management process. Hearing-aid-connected apps are increasingly common in audiological care, making it important to evaluate their availability and quality for clinicians, developers, and end-users. This scoping review aimed to identify, summarize, and synthesize information on clinically available hearing-aid-connected apps and evaluate their quality. **Methods**: A search of the Apple App Store (Canada) was conducted in August 2024 to identify current hearing-aid-connected apps that support hearing aid management. Metadata and features were extracted, and app quality was assessed using the Mobile Application Rating Scale (MARS). Quality was assessed across four objective domains (engagement, functionality, aesthetics, and information) and one subjective domain. **Results**: Apps had varying levels of metadata detail, including updates, compatibility, and target populations. All apps included common hearing aid controls (e.g., volume adjustment, microphone directionality), while more specialized features (tinnitus management, health tracking, remote clinician support) varied. High-performing apps scored significantly higher in engagement, functionality, aesthetics, and subjective quality, and all apps scored low in information quality, particularly for evidence and credibility. **Conclusions**: Findings highlight the need for transparent and informative metadata reporting and patient-centred design to improve clinical awareness, usability, and uptake of hearing-aid-connected apps.

## 1. Introduction

Hearing loss is the fourth leading cause of disability worldwide in terms of years lived with disability, affecting more than 20% of adult Canadians with diagnosed hearing loss [1,2]. When an individual with hearing loss uses a hearing aid, it can affect more than just their hearing ability; hearing aids have the potential to delay cognitive decline, reduce the risk of social isolation and depression, increase self-efficacy, and improve physical activity [3,4]. Despite the potential quality of life and health benefits associated with hearing aid usage, hearing aid uptake and adherence remain issues for many individuals [5]. Mobile health (mHealth) tools, such as smartphone apps, have the potential to support hearing aid adherence by providing person-centred hearing aid management. There are currently many apps available that are directed towards hearing aid users, and the literature has revealed that they offer valuable real-world insights into the early stages of a patient’s journey in improving their hearing health, as well as assisting with hearing aid management in the later stages [6]. Additionally, these apps are available across different stages and types of hearing care globally, though they are more commonly found in high-income countries [7]. Great potential exists regarding the implementation and effects of these apps, but further evaluation is needed to validate them.

Hearing-aid-connected apps allow for remote adjustment capability across a variety of hearing aid settings when compared to traditional hearing aid remote controls [8]. Apps are increasingly common, with most hearing aid manufacturers offering access to remote adjustment capability as part of their technology portfolio, involving the clinician and end-user/family. Numerous apps have been created to address other health issues, including diabetes, sleep management, smoking cessation, arthritis, and pain management, and are available for public use. Many of these healthcare apps have undergone review and quality assessment using standardized evaluation tools that were created for use with mHealth applications. One commonly used tool is the Mobile Application Rating Scale (MARS); this evaluation tool has been found to be an objective and easy-to-use tool that can classify and assess mHealth apps [9]. App evaluations can lead to recommendations for healthcare professionals, developers, and end-users regarding the quality of apps and their suggested uses. There is currently limited evidence describing the quality, content, and functionality of hearing-aid-connected apps, resulting in a gap in clinical guidance regarding the implementation and use of these apps. This study aimed to provide details on the current landscape of hearing-aid-connected apps through the completion of a systematic scoping review, including a quality assessment using the MARS tool.

## 2. Materials and Methods

A review of apps currently available through the Apple App Store for connecting and making changes to hearing aids was conducted following the Joanna Briggs Institute’s (JBI) methodology for scoping reviews, adapted for a review of apps [10]. This review aimed to identify the landscape of clinically available hearing-aid-connected apps, describe their characteristics, assess the objective quality of these apps using existing assessment tools, and provide recommendations for design and implementation to support hearing aid management. Data are reported using Preferred Reporting Items for Systematic reviews and Meta-Analyses extension for Scoping Reviews (PRISMA-ScR) guidance and checklist, Supplement S1 [11,12,13].

### 2.1. Protocol and Registration

The research team (DG, KP, RO) created a protocol document published via Open Science Framework (https://doi.org/10.17605/OSF.IO/SPTZN) before beginning the scoping review [14]. The protocol outlined the search terms, search strategy, eligibility criteria for apps to be included in the review, and steps for data extraction. Due to the use of secondary and anonymized information within this scoping review, ethics approval was not required [15]. The scoping review of the Canadian Apple App Store was conducted on 7 August 2024.

### 2.2. Eligibility Criteria

Apps were included in this review using the following inclusion criteria: (a) delivered content for hearing aid management for any age (beyond tracking apps, i.e., find my device); (b) paired with traditional hearing aid devices (clinic-based delivery model, excluding over the counter/personal sound amplification product & white-labeled devices); (c) have been updated within the last 2 years; (d) available in the English language; (e) designed for end-users in Canada; (f) free to download, not requiring in-app purchases; (g) the most current app and version per manufacturer; (h) compatible with iOS 17.6 (August 2024); and (i) offers clinician & end-user adjustment capability.

### 2.3. Information Sources

The Canadian Apple App Store was used to search for relevant apps on an iPhone 13. The apps had to be actively updated and compatible with iOS 17.7.1. The Apple App Store was selected for use, while the Google Play Store was used to compare and ensure all of the apps were also available for Android devices.

### 2.4. Search Terms

Three authors (KP, RO, DG) constructed a list of search terms. The Apple App Store was searched using the search terms “hearing aids” and “hearing aid app.”

### 2.5. Selection of Sources of Evidence

Apps were selected using a two-phase screening process. Phase 1 involved reviewing the App Store to determine if an app met the criteria for further assessment, which included examining the app’s title, description, and preview photos. All apps were recorded in a Word document. Phase 1 screening was completed by two independent reviewers (KP, RO) through a review of the results from the searches conducted in the App Store, comparing the results for each search term. Both reviewers have previous experience with hearing aids. KP is currently completing an Masters of Clinical Science degree in Audiology and has two years of hands-on experience as a research assistant. RO is a research associate with 7 years of experience working with hearing aids and hearing aid apps.

Once identified for inclusion in Phase 1, a research team member documented apps to be screened in full through App Store downloads. Apps identified as “include” or “maybe” were included for full review. Phase 2 included a full review of the selected apps against the inclusion criteria. Any difference in opinion over the eligibility of an app to be included in the review was addressed by DG during team meetings. Following the screening phases of the review, the research team further reviewed all eligible apps to assess app content availability. This step required pairing each app to a corresponding physical hearing aid. All hearing aid manufacturers with eligible apps were contacted to discuss the inclusion of their app based on the availability of loaner hearing aids to pair with the app.

### 2.6. Data Charting Process and Data Items

The research team created a data extraction table in Excel to collect information about the hearing aid app from the App Store (this information is referred to as metadata), as outlined in Appendix A, and to collate app evaluation data according to the Mobile Application Rating Scale (MARS) [9,16]. The MARS tool was converted into an online survey using Western University’s Qualtrics to ensure independent and blinded quality assessment of the apps [17]. The MARS was chosen over other standardized evaluation tools as it is one of the most frequently used tools in mHealth app quality evaluations [18]. Following a literature review of publicly available tools for assessing the quality of health mobile apps, the MARS was found to be multidimensional (encompassing a range of app quality indicators applicable to hearing aid research), objective (including the use of rating scales), quick to complete (a factor when considering its applicability to research with patients) and reliable (with published interrater reliability data). The MARS, a 23-item assessment tool, objectively assesses app quality over four dimensions (engagement, functionality, aesthetics, and information), and through questions assessing subjective app quality (Table 1) [9]. All questions were delivered using 5-point Likert scales, with descriptors provided across categories to guide app component review. The 5-point Likert scale rating explanations differed for each question, correlating with the question topic (e.g., Is the app fun/entertaining to use? 1—dull, not fun or entertaining at all, 2—mostly boring, 3—OK, fun enough to entertain user for a brief time (<5 min), 4—moderately fun and entertaining, would entertain user for some time (5–10 min total), 5—highly entertaining and fun, would stimulate repeat use); however, a rating of 5 was always the highest rating, while 1 was the lowest. Two reviewers (KP, RO) independently extracted data from each app. The app order was randomized for each reviewer, and the team met at regularly scheduled team meetings to reach a consensus. Consistent with the procedures described by Salzar and colleagues, discrepancies in scores between reviewers were examined. For any app where a substantial discrepancy was identified (defined as a difference greater than two points), the corresponding MARS items were jointly reviewed by at least two team members to determine consensus on one final rating (KP, RO, DG) [19].

### 2.7. Data Synthesis

Data from Qualtrics was downloaded from the online servers and copied into the main data extraction template in Excel. Analyses were conducted using both Excel and RStudio (v2024.12.0+467, Posit Software, PBC, Boston, MA, USA). Metadata, app features, and MARS data were collated for each app. Frequency counts were generated for app features including hearing aid adjustments, Bluetooth functionality, information, health monitoring, clinician support and hearing health. The average score between two individual raters was calculated for all MARS items and domains.

#### Statistical Methods

A descriptive analysis was performed on the data. The MARS scores for each section and the average app quality were calculated. The information score reflects accuracy, goal setting, quality and quantity of information, visual information, credibility, and excluded evidence base. The item for evidence base was removed from the analyses, following Mani and colleagues, as no apps were found to have efficacy studies completed [20].

App objective quality mean scores were calculated and separated into three performance categories: best-performing (range = 3.98–4.24), mid-performing (range = 3.73–3.3.97), and lowest-performing (3.47–3.72). A mean score was calculated for each app according to each corresponding section of the MARS and compared via analysis of variance (ANOVA). Across the three performance categories, the average score for each section of the MARS was calculated to compare overall performance in MARS sections via ANOVA. Tukey post hoc analyses were conducted within each MARS dimension identified as exhibiting significant differences between tertiles.

## 3. Results

### 3.1. Selection of Apps

**Figure 1:**
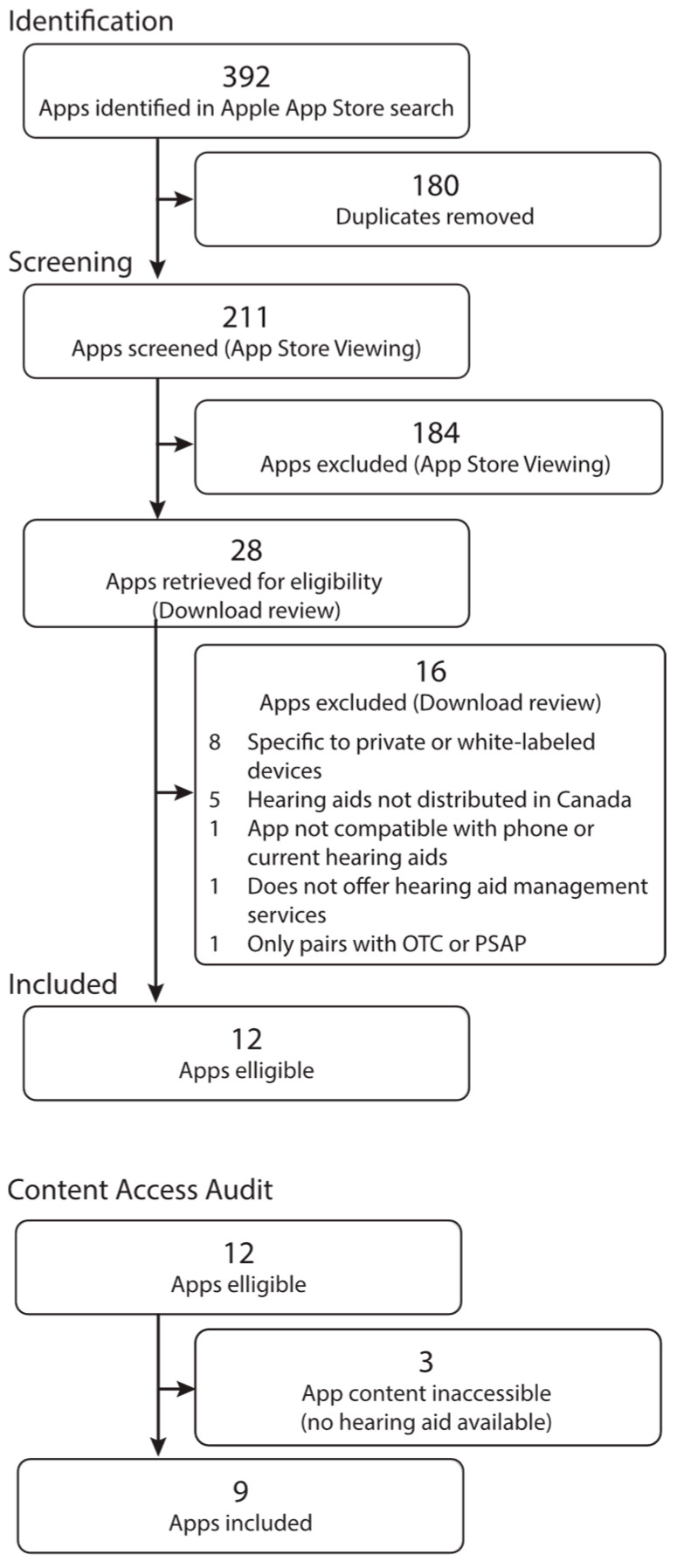
Process for App Inclusion, Including the PRISMA-ScR Flow Diagram.

During the initial search, 392 apps were identified, and duplicate apps were manually removed (*n* = 180), resulting in 211 apps being screened during phase 1. Twenty-eight apps were identified in Phase 1 and underwent additional screening. Of the apps identified for phase 2 screening, 16 were excluded based on the following criteria: the app was only compatible with specific or white-labeled devices (*n* = 8), the hearing aids were not distributed in Canada (*n* = 5), the app was not compatible with the phone model, iOS 17.7.1 or current hearing aids (*n* = 1), the app did not offer hearing aid management services (*n* = 1), and the app only paired with over the counter or personal sound amplification products (*n* = 1). A total of 12 apps met the eligibility criteria for this study, as illustrated using the PRISMA-ScR flow diagram (Figure 1). As an additional step, a content access audit was conducted to determine whether the research team had access to the content necessary for data extraction. This audit further screened the apps to assess whether the full content could be accessed (i.e., by pairing loaner hearing aid devices to the app). This audit identified three apps in which the content was inaccessible.

### 3.2. Metadata

Metadata are reported for all apps and according to their respective App Store details (Appendix B). All apps had been updated within the 6 months before data collection, with most apps undergoing an update within the last 3 months (*n* = 8). App Store data included iOS ratings, with an average Apple rating of 3.6 stars (range: 1.6–4.7) and a wide range in the number of ratings per app (range: 8–6600). Six apps were identified in the App Store as rated for users aged 4 years or older, one app was rated for users aged 12 years or older, and the remaining apps were rated for users aged 17 years or older; however, only one app has been approved as a pediatric-focused app in Canada. Additionally, five apps identified compatibility with one or more hearing aid platforms; four apps did not include device compatibility details in the App Store. App updates were reported through the App Store when available.

### 3.3. App Features

**Figure 2:**
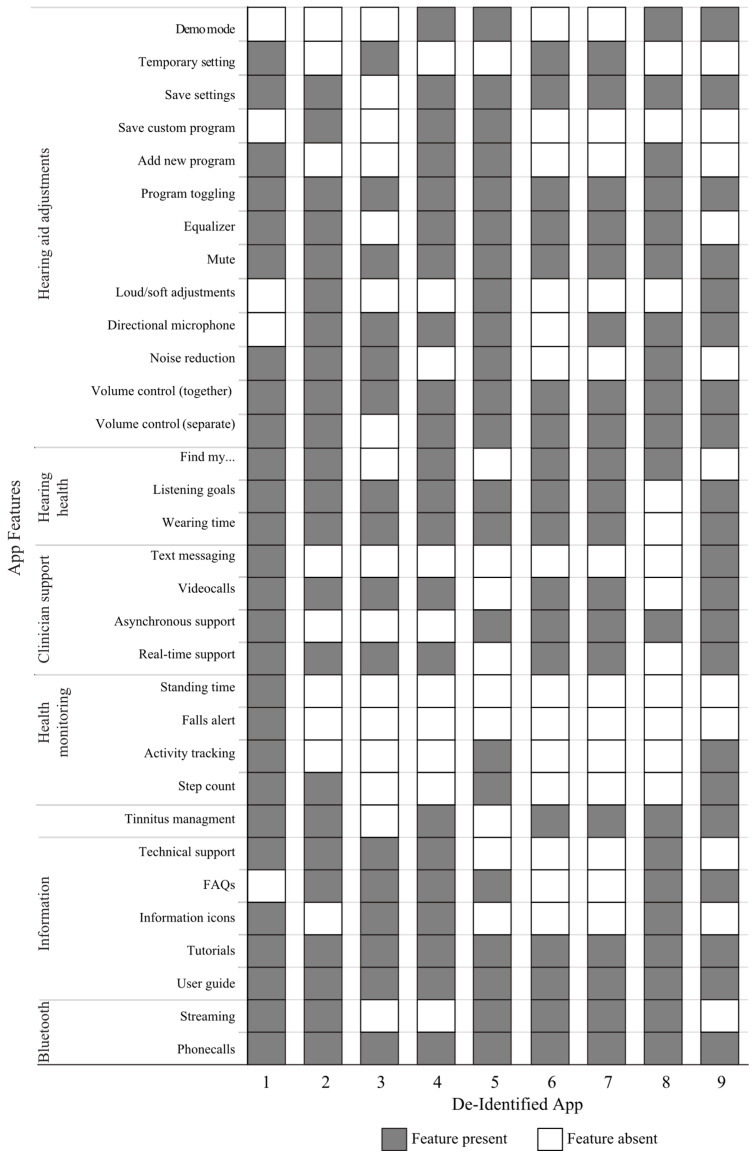
Summary of App Features.

The app features explored in this review included end-user controls, program management, Bluetooth functionality, user support, tinnitus management, health tracking, remote support, and any additional factors identified in each app. A total of 32 features were identified in this review. No single app contained all these features. Figure 2 outlines the functionality of each app, including the end-user controls available in each app. All apps allow the end-user to make direct changes to their hearing aids in the moment using a smartphone. All apps included the ability to change the directional microphone, the option to mute the hearing aids, and the ability to adjust the volume of the hearing aids as a pair. The ability to save settings to any program was limited to only one of the apps. If you exited and closed the app from running in the background, the settings would not be saved. However, if the app was left open in the background, any adjustments made would be retained. All apps included at least one component of hearing health features (e.g., setting listening goals), and five included all available components in this category. The least frequently included feature was specific to the adjustment of loud or soft sounds (*n* = 3). Health monitoring was the least frequently included category of features in the apps, with only four apps featuring any health monitoring component. This meant that these apps could track one or more of the following: step count, activity tracking, fall alerts and/or stand time. One manufacturer used two apps to enable the end-user hearing aid adjustments and clinician support, whereas all other apps combined both categories of features into a single app. All apps included some form of in-app or web-based tutorials and user guides, whereas information icons, frequently asked questions, and technical support options varied across the apps. All apps included at least one method for connecting the end-user to their clinician for support, varying in terms of synchronous versus asynchronous connections and text-based versus video-based connections. Reported Bluetooth compatibility indicated that all apps could connect audio for phone calls, with six apps indicating connections for streaming programs. Two different manufacturers’ apps had identical interfaces and reported the same developer.

### 3.4. Quality Evaluation

For all included apps, average scores were calculated for each of the four MARS objective quality dimensions and averaged together to produce the overall quality score (Table 2). Four items were identified as having a significant difference between raters and underwent consensus by the research team. Once the four items underwent consensus, the research team then conducted analyses of the quality ratings. Of the four objective quality dimensions, information had the least variance in ratings (0.58), whereas engagement had the highest variance (1.55) and contained the lowest rating (2.60). No app received a perfect score (5.0) for any objective quality rating category; however, four apps received an individual score of 5 for five separate items: one app for interactivity and quantity of the information, one app for both the performance and accuracy of the app description, one app for visual appeal and quantity of the information, and one app for only the quantity of the information.

Subjective scores ranged from 2.88 to 5.00, with the lowest average rating across all apps for the question on what star rating the raters would give (average score 3.33). The remaining subjective quality ratings were higher, with a 4.33 rating for recommending the app to others, 4.72 for frequency of use, and 4.44 for willingness to pay for the app. Average app subjective quality ratings were higher than objective ratings (subjective quality = 4.21, objective quality = 3.86).

**Figure 3:**
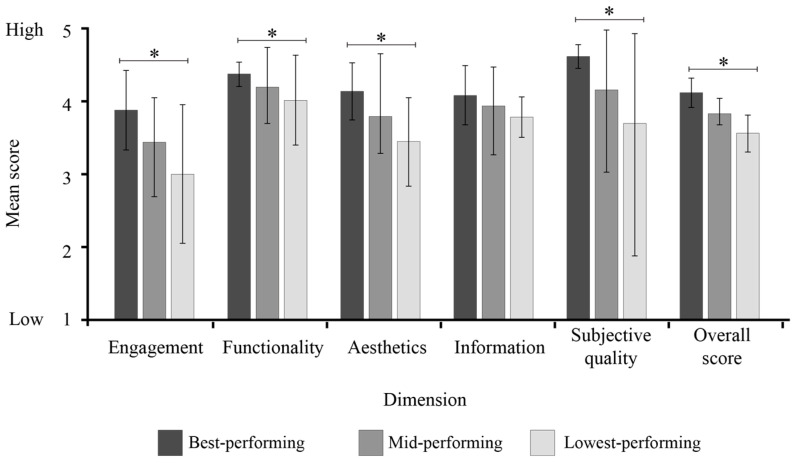
Comparison of Tertile Ratings Across MARS Dimensions. * Indicates significant differences between corresponding tertile categories at the *p* = 0.05 level for ratings within the respective category.

Mean ratings and associated confidence intervals were calculated for the tertiles for each MARS dimension and compared using ANOVA and Tukey post hoc comparisons (Figure 3). Significant differences were not identified when comparing ratings across the MARS domains. ANOVA analyses revealed significant differences between tertiles within the functionality domain (F(2, 6) = 5.56, *p* = 0.043), the aesthetics domain (F(2, 6) = 5.56, *p* = 0.043), the subjective quality domain (F (2, 12) = 3.966, *p* = 0.048), and the overall quality domain (F (2, 12) = 4.262, *p* = 0.040).

Completion of post hoc analyses revealed that the best-performing tertile was rated significantly higher than the lowest-performing apps for the overall quality score (*p* = 0.032), subjective quality (*p* = 0.039), engagement (*p* = 0.002), functionality (*p* = 0.014) and aesthetics (*p* = 0.043). No significant differences were identified between the mid-performing and best-performing or the mid-performing and lowest-performing tertiles across all the domains.

## 4. Discussion

This scoping review aimed to identify the available hearing-aid-connected apps in the Canadian Apple App Store and evaluate their quality and functionality, with the end goal of providing recommendations for healthcare professionals, app developers, and end-users regarding app quality and suggested use. To our knowledge, this study is the first to evaluate hearing-aid-connected apps using the MARS evaluation tool to provide quality indicators across engagement, functionality, aesthetics, information quality and subjective quality. Before discussing the apps, it is essential to acknowledge that the mobile app market is rapidly evolving and unpredictable; the state of the market at the time of this publication may differ from what is described in this review.

### 4.1. MARS Quality Ratings

The overall mean engagement values for the high-performing apps (*n* = 3) were significantly different from those of the low-performing apps, suggesting that these apps were more entertaining, engaging, and potentially more interactive. The MARS also investigates customization; a study by Bol and colleagues explored the importance of customization in mHealth apps, specifically those related to physical activity [21]. They found that individuals with a stronger need for autonomy were more likely to engage in the desired app activities when given the opportunity to customize the app. The hearing-aid-connected apps, specifically high-performing apps, give users who are seeking more personalized and autonomous control over their hearing aid usage a way to do so. Having the option for customization within hearing-aid-connected apps is an important aspect for user engagement. Another component of app engagement is whether the app is designed in such a way that it targets the right demographic. A study by Chatzipavlou and colleagues looked at establishing guidelines for developing mHealth Apps and outlined that app developers often face the challenge of understanding the overall market and the specific users their apps are designed for [22]. Our current study demonstrates that developers can struggle and have general challenges in meeting the goals of an app targeted to the many different age ranges and digital literacy levels of hearing aid users. Future app development should focus on ensuring that hearing-aid-connected apps meet the needs of a wide range of target audiences, which is essential for app effectiveness. When an app is designed to meet older adults’ needs, it has the potential to be a suitable tool for disease prevention, lifestyle changes, and health management [23]. For example, hearing-aid-connected apps allow end-users to set listening goals regarding how long they will wear their hearing aids each day. Their audiologist also has access to the patient’s hearing aid wear time through the app’s metadata to help keep the patient on track regarding their listening goals. As a team, this process enables the patient and audiologist to collaborate on increasing their hearing aid usage, ultimately benefiting their overall health and well-being. Therefore, when hearing-aid-connected apps are designed appropriately for their target groups, they have the potential to facilitate better hearing aid usage and, overall, more favourable hearing health.

The mean values of the high-performing apps within the MARS functionality section were significantly different from those of low-performing apps, suggesting that high-performing apps may be easier for end-users to use and navigate. A study by Ramdowar and colleagues found that simple navigation systems should be employed within mHealth apps for the older adult population to reduce user interface challenges [24]. For example, ensuring font, button, and icon sizes are clear, using high contrast colours, and providing clear instructions when learning to navigate an app. It’s essential to balance the needs of the target population with the ease of use of all hearing-aid-connected apps to deliver the optimal experience for the end-user. In addition to the functionality scores focusing on the needs of the target population and ease of use, aesthetics also focuses on similar aspects. The mean values of the high-performing apps within the aesthetics section of the MARS were significantly different from those of the low-performing apps. High-performing apps often feature more visually appealing elements, including high-quality graphics and intuitive buttons. Lazard and colleagues found that in order to increase mHealth app adoption, it is essential to understand the target population’s expectations regarding app features’ appearance [25]. However, Rowland and colleagues found that end-users are not often involved in the development process of mHealth applications, and the development of clinically focused apps may not reflect the end-users’ needs or desires [26]. A study by Glista and colleagues integrated participatory research methods with audiologists to inform the future design and clinical use of adolescent-centred mHealth applications; findings from this work highlight clinical implementation consideration for hearing-aid-connected apps as part of a collaborative care model (i.e., involving adolescent-clinician interaction in the process) [27]. This work highlights the benefit of including end-user feedback as part of the app design process.

The information dimension included in the quality assessment was the only component where the high-performing app category mean was not statistically significant compared to the mid- or low-performing app categories. Additionally, the scores for this section were low overall. This finding may suggest that credibility, availability, and source of information for these hearing-aid-connected apps are lacking. Specifically lacking was the evidence base subsection within the information section. This subsection looked at whether the app had been trialed/tested and then published in the literature. Future research and publicly available data can enhance the information availability and credibility, thereby expanding the research knowledge base on the benefits of app use. The addition of this research base in the future could help support both clinicians and end-users’ choice of incorporating mHealth app use into their hearing healthcare.

The goal of the MARS rating scale is to classify and assess the quality of mHealth apps through a simple, objective, and reliable tool. It is understood that the individual providing the ratings may bring their own biases or influences on their subjective app ratings found in the App Store, and can vary significantly [9]. Upon completing the objective section of the MARS, raters complete a similar star rating. Stoyanov and colleagues explain that upon completing the entire MARS, the star rating likely provides a more reliable subjective measure of overall app quality [9]. The data found in this review supports this finding, where the evaluators’ star ratings decrease similarly to the objective quality score between the high-performing and low-performing apps.

### 4.2. Summary of Included Apps and Key Study Findings

The App Store age classifications analyzed in this review do not necessarily coincide with the recommended population/age range found in the app’s manual. Clinicians should advise clients that certain age groups may be directed to use the app based on the manufacturer’s documentation, rather than relying on App Store information. The research team observed inconsistent use of terminology across the apps when defining or describing app features. A standardized list of features provided by the app manufacturer would be beneficial for clinicians, as illustrated in Figure 2. Incorporating consistent terminology would aid clinicians who work with different hearing aid manufacturers in better guiding and supporting patients’ use of apps and app features. Each app, except for the pediatric app, was created by a different hearing aid manufacturer; however, it is common for manufacturers to operate within the organizational framework of their parent corporations. In this study, two apps owned by the same parent corporation had similar interfaces, with minor differences, including the colour scheme to match their branding.

The key findings from this study suggest that hearing-aid-connected apps are clinically available to support most current hearing aid models. This review highlights the variability in feature options accessible to end-users, ranging from adjustment features that are universally accessible across all apps (e.g., volume control, mute options, and program toggling) to more specialized features (e.g., tinnitus management and health monitoring). High-quality ratings revealed usability strengths related to engagement, functionality, and aesthetic components. More comprehensive and consistent reporting and design practices, including improved metadata reporting and patient-centred design practices, will enhance usability, clinical integration, and uptake of these tools.

### 4.3. Future Directions

The research team would like to provide future design considerations to improve app engagement and ensure high-quality app information, as a result of this review. These include integrating in-app feedback, such as notifications to support goal attainment (e.g., daily hearing aid use time goals). Enabling personalized content to engage the user, such as tailored support materials (e.g., age-specific and/or unique to user goals). Initiating peer-to-peer support mechanisms and connection to hearing healthcare providers, such as messaging via a social community and data sharing with the provider. Tailoring the app to align with digital literacy and mHealth readiness, including shared decision-making between the patient and their hearing care provider and adopting a standardized app features reporting method for clinical consumption. Manufacturers should provide clear explanations to clinicians and end-users regarding app updates and the details around the changes made to the functionality, to ensure smooth transitions between updates, beyond the explanations in the App Store. Finally, app developers could incorporate user/provider feedback as part of the design process and consider universal design principles to facilitate improved interface accessibility. Findings from a recent focus group study, including adolescent-centred mHealth, aligned with many of the future recommendations mentioned here [27]. For example, clinicians reported the value of integrating end-user feedback around hearing aid personalization (or tailoring the app to individual interests and needs), indicating this step as important to the design and resulting functionality of mHealth apps. This research highlights the importance of integrating diverse perspectives in the development and evaluation of advanced technologies in hearing healthcare.

### 4.4. Limitations

The findings of this review must be considered within the context of study limitations. The two researchers who reviewed the apps were young, female, non-hearing aid users who were familiar with this type of technology. Generalizability of results could be improved by involving reviewers who are more closely aligned with the apps’ primary target audiences. Additionally, while this review only investigated how hearing-aid-connected apps function on Apple iPhones due to popularity and accessibility, Android products were not investigated; future investigations into how all apps function across smartphone platforms are warranted. Furthermore, this review only investigated hearing-aid-connected apps that were available in Canada. For the findings to apply to an international setting, further investigation would need to be done outside of Canada.

Throughout the study, a handful of the downloaded apps underwent manufacturer updates, which were automatically installed on the lab iPhones. The research team was initially unaware of the updates; once it became clear that apps were undergoing automatic updates, the research team changed the Apple Store settings to prevent automatic updates. A member of the team then went through all downloaded apps in the App Store to identify apps that had been recently updated, and these apps were then re-screened by two members of the team for any updates to the MARS scores and metadata. Time stamps were added to the evaluations to be consistent within and across apps. Future app-based scoping reviews should ensure that App Store or Play Store updates are turned off to ensure consistent version codes across evaluators.

Apps were initially enabled within the fitting software, including a default assortment of features and settings. Some specialized app features, such as tinnitus management, could only be accessed after being enabled through the fitting software. The research team discovered this setting discrepancy after the initial evaluations were completed, resulting in the research team re-programming hearing aids with this optional feature and re-completing the evaluations. Future studies should ensure that all relevant features are activated within the programming interface before beginning the evaluations. Additionally, future research should be done to inform end-users about the possible features that are available to them in these kinds of apps and the path that needs to be taken to access them.

Notably, the consistently low scores in the information domain of the evaluation tool, coupled with the exclusion of the evidence-based item from the final scoring criteria, underscore a critical gap; there is a paucity of peer-reviewed clinical trials or supporting research literature on hearing-aid-connected apps. This finding is unique to hearing healthcare apps, when compared to other allied health fields, where there is a larger evidence base to support access to information, credibility, and quality of information. This limitation highlights the need for further empirical research to support the clinical use of such apps.

## 5. Conclusions

This scoping review is the first to report on the scope and quality of hearing-aid-connected apps using an established quality evaluation tool (the MARS framework). Quality varied across engagement, functionality, aesthetics, and information of the reviewed apps. High-performing apps offered the highest scores for user engagement and ease of use; all apps had low scores in the information domain, highlighting a lack of supporting evidence. The findings of this scoping review emphasize the need for improved app design, standardized feature reporting, and greater transparency in updates. Clinicians should be aware of variability across apps when establishing patient use recommendations, and developers must consider digital literacy and the needs of a diverse range of end-users in the design of future apps. Further research is essential to build an evidence base for hearing-aid-connected apps and to support their integration into hearing healthcare. With thoughtful improvements, hearing-aid-connected apps hold strong potential to further enhance the hearing aid user experience, across all ages, supporting improved engagement, treatment satisfaction, and empowering individuals in their hearing healthcare.

## Figures and Tables

**Table 1:** Mobile Application Rating Scale (MARS) Structure.

Dimension	Items
Objective Quality	
Engagement	Entertainment, interest, customization, interactivity, target group
Functionality	Performance, ease of use, navigation, gestural design
Aesthetics	Layout, graphics, visual appeal
Information	Accuracy, goals, quality, quantity, visual information, credibility, evidence-based
Subjective Quality	Personal interest level of the app (recommendation of the app to others, how often they would use it, if they would pay for the app)
Evaluator Star Rating	The overall star rating the individual would give the app

**Table 2:** MARS Mean Scores Across Apps.

Category	App	Overall Quality	Objective Quality Dimensions				Objective Quality	Subjective Quality	Evaluators’ Star Rating
			Engagement	Functionality	Aesthetics	Information			
High-performing	1	4.49	4.15	4.38	4.17	4.25	4.24	4.75	4.00
	2	4.38	3.70	4.38	4.33	4.17	4.14	4.63	3.50
	3	4.35	3.85	4.50	4.00	3.92	4.07	4.63	3.50
Mid-performing	4	4.09	3.35	4.38	3.83	4.17	3.93	4.25	3.50
	5	4.33	3.67	4.00	4.33	3.67	3.92	4.75	4.00
	6	3.77	3.10	4.38	3.83	3.83	3.79	3.75	3.00
Low-performing	7	4.03	2.95	4.25	3.67	3.83	3.68	4.38	3.00
	8	3.71	3.40	3.75	3.17	3.90	3.55	3.88	3.00
	9	3.17	2.60	4.13	3.50	3.67	3.47	2.88	2.50
Mean		4.04	3.42	4.24	3.87	3.93	3.86	4.21	3.33

## Data Availability

The data presented in this study are available on request from the corresponding author due to privacy reasons.

## Supplementary Materials

The following supporting information can be downloaded at: https://www.mdpi.com/article/10.3390/audiolres15060157/s1. Table S1. Preferred Reporting Items for Systematic Review and Meta Analysis extension for Scoping reviews (PRISMA-ScR) Checklist.

## Abbreviations

The following abbreviations are used in this manuscript:

MARS	Mobile Application Rating Scale
PRISMA-ScR	Preferred Reporting Items for Systematic reviews and Meta-Analyses extension for Scoping Reviews

## Appendix A

**Table A1:** Data Extraction Components.

Dimension	Items	Response Options
Metadata	Manufacturer	Open-text
	iOS rating	Open-text
	Age classification	Open-text
	App version	Open-text
	Date of last update	Open-text
	Data privacy	
	Analytics	Health and fitness, location, identifiers, usage data, diagnostics, and other data
	App functionality	Health and fitness, location, identifiers, usage data, contact information, diagnostics, and other data
	Other purposes	Contact information
	Privacy notice	Yes/No
	User manual	Yes/No
App features	Remote control	
	Volume	Yes/No
	Mute	Yes/No
	Noise reduction	Yes/No
	Directional microphone	Yes/No
	Loud/soft adjustments	Yes/No
	Equalizer	Yes/No
	Program toggling	Yes/No
	Program management	Yes/No
	Bluetooth functionality	Yes/No
	User supports	Yes/No
	User guide	
	Tutorials/videos	Yes/No
	Information icons	Yes/No
	Technical support	Yes/No
	Tinnitus management	Yes/No
	Health tracking	
	Step count	Yes/No
	Activity tracking	Yes/No
	Falls alerts	Yes/No
	Stand time	Yes/No
	Remote support	
	Synchronous support	Yes/No
	Asynchronous support	Yes/No
	Video	Yes/No
	Messaging	Yes/No
	Other	
	Wearing time	Yes/No
	Listening goals	Yes/No
	Notifications & reminders	Yes/No
	Find my hearing aids	Yes/No
	Remote connection	
	Does the app allow the user to connect with their hearing healthcare professional?	Yes/No

## Appendix B

**Table A2:** App Store Metadata. In alphabetical order according to App Name.

App Name (Manufacturer)	Developer	iOS App Rating (Number of Ratings)	Age	App Version	Date Last Updated ^1^	Data Linked to End-User ^2^	Data Not Linked to End-User ^2^
Bernafon App (Bernafon)	Bernafon AG	1.6 (8)	4+	1.3.1	3 months	None	Analytics: Identifiers (user ID, device ID), usage data (product interaction) App functionality: Location (coarse location), contact info (email address), identifiers (user ID), diagnostics (crash data, performance data)
myPhonak (Phonak)	Sonova AG	4.4 (6600)	17+	6.8.0	1 day	None	Analytics: Identifiers (device ID); usage data (product interaction); diagnostics (crash data, performance data) App functionality: Identifiers (device ID); usage data (product interaction); diagnostics (crash data, performance data)
myPhonak JR (Phonak)	Sonova AG	4.4 (33)	4+	1.3.0	5 months	None	Analytics: Identifiers (device ID); usage data (product interaction); diagnostics (crash data, performance data) App functionality: Identifiers (device ID); usage data (product interaction); diagnostics (crash data, performance data)
My Starkey (Starkey)	Starkey Laboratories	3.8 (9)	12+	2.1.0	2 months	Analytics: Health and fitness App functionality: Health and fitness, contact info (email address, name)	Analytics: Usage data (product interaction); diagnostics (performance data) App functionality: Location (precise location), usage data (product information), diagnostics (crash data, performance data, other diagnostic data)
Oticon Companion (Oticon)	Oticon A/S	2.2 (81)	4+	1.3.1	3 months	None	Analytics: Identifiers (user ID, device ID), usage data (product information) App functionality: Location (coarse location), contact info (email address), identifiers (user ID), diagnostics (crash data, performance data)
Resound Smart 3D (ReSound)	ReSound	4.5 (2200)	4+	1.35.1	4 months	None	Analytics: Health & fitness (health), identifiers (user ID, device ID), usage data (product interaction), sensitive info (sensitive info), diagnostics (crash data); app functionality (user content (customer support), diagnostics (crash data)
Signia App (Signia)	Sivantos Pte. Ltd.	4.1 (839)	17+	2.6.71	3 months	Analytics: Identifiers (user ID) App functionality: Identifiers (user ID)	Analytics: Usage data (product interaction); diagnostics (crash data; performance data; other diagnostic data) App functionality: Usage data (product interaction)
Unitron Remote Plus (Unitron)	Sonova AG	4.4 (309)	17+	5.1.0	2 months	Analytics: Other data App functionality: Other data, other (contact info-email address & name)	Analytics: Usage data (product interaction), diagnostics (crash data, performance data)
Widex Moment (Widex)	Widex A/S	3.2 (43)	17+	1.8.0	6 months	Analytics: Health and fitness (health), location (coarse location), user content (other user content), identifiers (user ID, device ID), usage data (product interaction, other usage data), diagnostics (crash data, performance data), other data (other data types) App functionality: Diagnostics (crash data)	None

Note. ^1^ Date of last update, as reported in the App Store on 7 August 2024. ^2^ Data as reported in the App Store.
